# Ossification of the acetabular rim: a highly prevalent finding in asymptomatic non-osteoarthritic hips of all ages

**DOI:** 10.1007/s00330-021-07750-y

**Published:** 2021-03-13

**Authors:** Catarina Valente, Laura Haefliger, Julien Favre, Patrick Omoumi

**Affiliations:** 1 Department of Radiology and Interventional Radiology, Tours University Hospital (CHRU) and University of Tours, 37000 Tours, France; 2 Department of Diagnostic and Interventional Radiology, Lausanne University Hospital (CHUV) and University of Lausanne (UNIL), Rue du Bugnon 46, 1011 Lausanne, Switzerland; 3 Department of Musculoskeletal Medicine (DAL), Swiss BioMotion Lab, Lausanne University Hospital (CHUV) and University of Lausanne (UNIL), 1011 Lausanne, Switzerland; 4 Department of Radiology, Cliniques Universitaires St Luc - UC Louvain, Brussels, Belgium

**Keywords:** Hip joint, Heterotopic ossification, Osteophyte, Osteoarthritis, Femoroacetabular impingement

## Abstract

**Objective:**

To estimate the prevalence of acetabular rim ossifications in the adult population with asymptomatic, morphologically normal hips at CT and to determine whether the presence of these ossifications is associated with patient- or hip-related parameters.

**Methods:**

We prospectively included all patients undergoing thoracoabdominal CT over a 3-month period. After exclusion of patients with a clinical history of hip pathology and/or with signs of osteoarthritis on CT, we included a total of 150 hips from 75 patients. We analyzed the presence and the size of ossifications around the acetabular rim. The relationships between the size of acetabular rim ossifications and patient-related (sex, age, BMI) or hip-related parameters (joint space width, and cam- and pincer-type femoroacetabular impingement morphology) were tested using multiple regression analysis.

**Results:**

The prevalence of acetabular rim ossifications in this population of asymptomatic, non-osteoarthritic hips was 96% (95% CI = [80.1; 100.0]). The presence of ossifications and their size were correlated between the right and left hips (Spearman coefficient = 0.64 (95% CI = [0.46;0.79]), *p* < 0.05)). The size of acetabular rim ossifications was significantly associated with age (*p* < 0.0001) but not with BMI (*p* = 0.35), gender (*p* = 0.05), joint space width (*p* ≥ 0.53 for all locations), or any of the qualitative or quantitative parameters associated with femoroacetabular morphology (*p* ≥ 0.34).

**Conclusion:**

Acetabular rim ossifications are highly prevalent in asymptomatic, non-osteoarthritic adult hips at all ages. Their size is not correlated with any patient- or hip-related parameters except for age. These findings suggest that ossifications at the acetabular rim, when present in isolation, should not be considered a sign of osteoarthritis or femoroacetabular impingement morphology.

**Key Points:**

*• Acetabular rim ossifications are extremely common in asymptomatic, non-osteoarthritic adult hips.*

*• Acetabular rim ossifications are present independently from other signs of osteoarthritis in adult hips at all ages and should not be interpreted as a pathological finding.*

*• The diagnosis of osteoarthritis or femoroacetabular impingement morphology should not be made based on the sole presence of ossifications at the acetabular rim.*

## Introduction

In clinical practice, ossifications adjoining the acetabular rim are frequently seen in otherwise healthy-appearing hip joints, at all ages. The origin of these acetabular rim ossifications (ARO) is unclear. While marginal osteophytes are classically found at this location, some authors have advocated that these ossifications may correspond to ossifications of the acetabular labrum, based on imaging and histological analyses [1–5]. At cross-sectional imaging, ARO are found at the attachment site of the labrum onto the acetabular rim and larger ossifications tend to have the same triangular shape as the labrum. At histology, a few studies have attempted to determine the origin of these ossifications, but the topic remains debated. While some authors believe that these ossifications correspond to an early stage of the formation of acetabular osteophytes, others describe a phenomenon distinct from osteophyte formation, either by endochondral ossification of the labrum or by appositional bone formation in the subperiosteal part of the acetabular rim, which displaces and replaces the labrum [1, 3, 6, 7].

The interpretation of these ossifications is also largely debated. While ARO have been described by some authors as variants that may mimic marginal acetabular osteophytes that can falsely lead to the diagnosis of hip joint osteoarthritis, others have hypothesized that ARO may be the cause or the consequence of femoroacetabular impingement (FAI) [1, 4, 5, 7].

Overall, the literature on ARO is limited, in particular in the healthy population, and their characteristics and origin not fully understood.

In this study, we aimed to estimate the prevalence of ARO in the adult population with asymptomatic, morphologically normal hips, using computed tomography (CT). We further aimed to determine whether the presence of ARO is associated with patient-related (sex, age, BMI) or hip-related parameters (joint space width (JSW) and imaging parameters of FAI morphology).

## Material and methods

### Population

This study was approved by the ethical committee of the institution where the data was acquired. Informed consent was obtained from all patients. Over a 3-month period, adult patients who underwent thoraco-abdomino-pelvic CT for suspected thoracic, abdominal, or urogenital pathology were prospectively included. All patients agreeing to participate in the study were asked to fill out a questionnaire enquiring about any current or past hip/groin pain, medical/surgical hip joint history, and history of hip trauma or history of hip problems during childhood. All patients who answered positively to any of the questions were excluded. Based on the analysis of CT examinations performed by a musculoskeletal radiologist with 3 years of experience (and who did not participate in the readings described below), we excluded all osteoarthritic hips, as defined by the presence of any of the following signs: joint space narrowing, osteophytes at other locations in the joint (marginal osteophytes of the femoral head, of the fovea capitis, or at the inner margin of the acetabulum, without considering osteophytes at the acetabular rim), or subchondral bone changes, including sclerosis or cysts. These abnormalities were interpreted according to the revised OARSI atlas of radiographic features in osteoarthritis [8]. In order to increase our sensitivity for the detection of joint space narrowing, we also excluded any patients with significant asymmetry between hips [9, 10]. Other exclusion criteria at CT included evidence of hip cartilage calcium deposition disease, posttraumatic deformity, os acetabuli, Legg-Calvé-Perthes disease, osteonecrosis, slipped capital femoral epiphysis, or hip dysplasia.

**Fig. 1:**
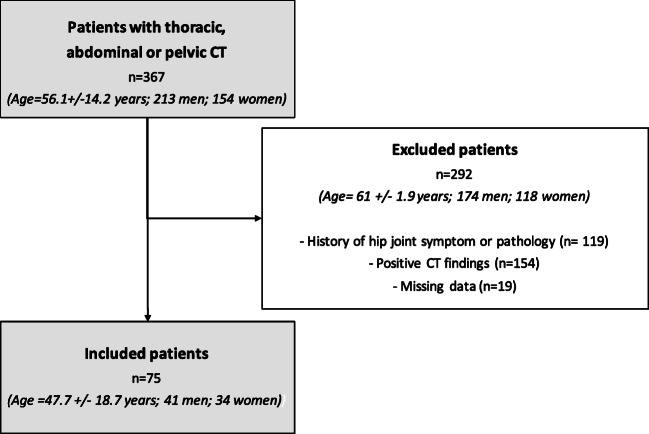
Flowchart shows selection criteria and patient characteristics

Figure 1 shows selection criteria and patient characteristics. Of all the patients who underwent a qualifying CT examination, 368 agreed to be part of the study. A total of 119 patients were excluded due to at least one positive answer to the questionnaire, 155 due to the presence of osteoarthritis or other hip pathologies at CT, and 19 patients were excluded because of missing data. In total, 150 hips of 75 patients were included in the study (41 men and 34 women), with a mean age of 47.7 years ± 18.8, and mean BMI of 24.29 kg/m2 ± 4.4.

### CT examination

CT examinations were performed on a 40-, 64-, or 256-MDCT scanner (Brillance 40, Brillance 64, and ICT 256, respectively, Philips Healthcare Inc.). CT data acquisition included coverage of the whole pelvis through the ischial tuberosities, using the same parameters (tube voltage = 120 kVp, reference tube current–time product = 90–200 mAs, automated dose modulation). The examination protocol was adapted whenever necessary to ensure that all examinations included a series of images at high-resolution, reconstructed using a bone algorithm, which was used for image analysis. CT examinations were stored on the Picture Archiving and Communication System (PACS) (Vue PACS, Carestream Health Inc.).

### Image analysis

The image analysis was performed by a musculoskeletal radiologist with 10 years of experience. A board-certified radiologist with 1 year of experience in musculoskeletal imaging read a subset of 80 hips from 40 patients to test interobserver agreement. Prior to the study, a training session on a selection of five hips which were not part of the study was performed by the two observers conjointly.

**Fig. 2:**
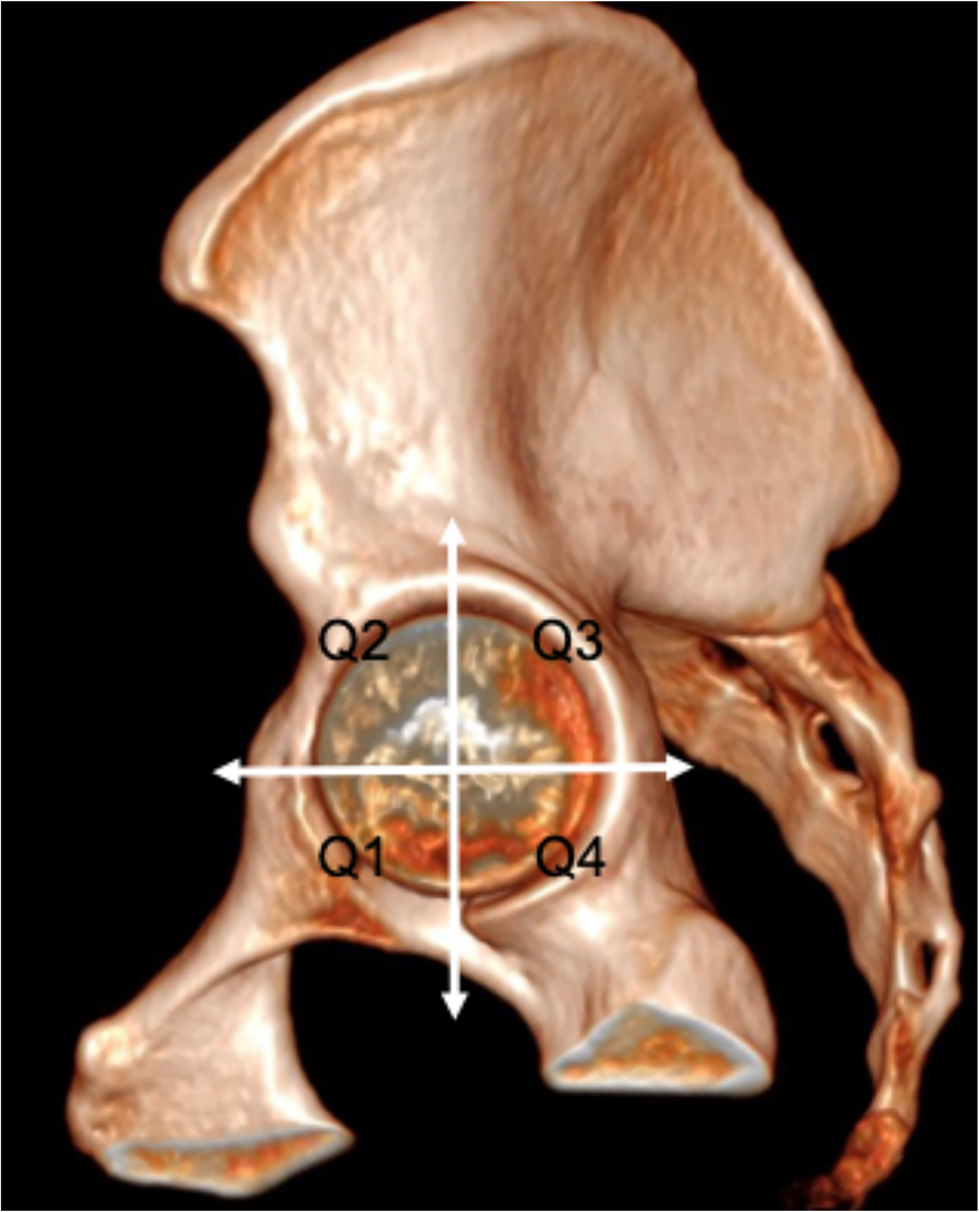
Volume rendering reconstruction of CT of left hip joint showing the location of the quadrants around the acetabular rim. (Q1) Anteroinferior quadrant, (Q2) anterosuperior quadrant, (Q3) posterosuperior quadrant, and (Q4) posteroinferior quadrant

**Fig. 3:**
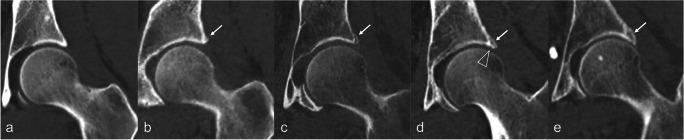
Coronal reformats of CT examinations of five hips showing different sizes of acetabular rim ossifications in the posterosuperior quadrant (arrows). **a** No ossifications (**b**–**e**) acetabular rim ossification with increasing size from left to right. Note the double-rim sign, which has been described for the diagnosis of labral ossification (two lines are visible at the location of the ossification, formed by the contour of the acetabular rim and that of the ossification). Also note a small notch between the acetabulum and the ossified labrum in **d**, likely corresponding to the chondrolabral recess, which has been described as a diagnostic clue to differentiate labral ossifications from osteophytes [4]

Image analysis was performed on a PACS workstation. Multiplanar reformats were generated by the readers. In order to detect and measure ARO, the acetabular rim was divided into four quadrants (anteroinferior (Q1), anterosuperior (Q2), posterosuperior (Q3), and posteroinferior (Q4)), according to the largest diameters of the femoral head in the coronal and axial reformats (Fig. 2). In each quadrant, the presence of ARO was assessed using the double-rim sign, where the acetabular rim is doubled by the contour of the ossification [1, 11] (Fig. 3). ARO were then measured (largest mediolateral diameter). If more than one ossification was present per quadrant, the largest was considered for analysis.

Quantitative parameters reported to be associated with FAI were measured on CT multiplanar reformats following methods previously described in the literature [12, 13]. Quantitative parameters associated with cam-type femoroacetabular impingement included the alpha angle measured anterosuperiorly at 45° angle and the femoral head-neck offset, while parameters associated with pincer-type FAI included the acetabular version angle, the lateral center-edge angle, and the acetabular index [12, 13].

Qualitative parameters reported to be associated with FAI were measured on a 300-mm-thick coronal multiplanar reconstruction with pixel intensity averaging, where the tip of the coccyx projected on the midline at approximately 2 cm above the upper border of the pubic symphysis. This reconstruction was used as a simulation of a pelvic radiograph that allowed the assessment of the crossover sign and the posterior wall sign [12].

**Fig. 4:**
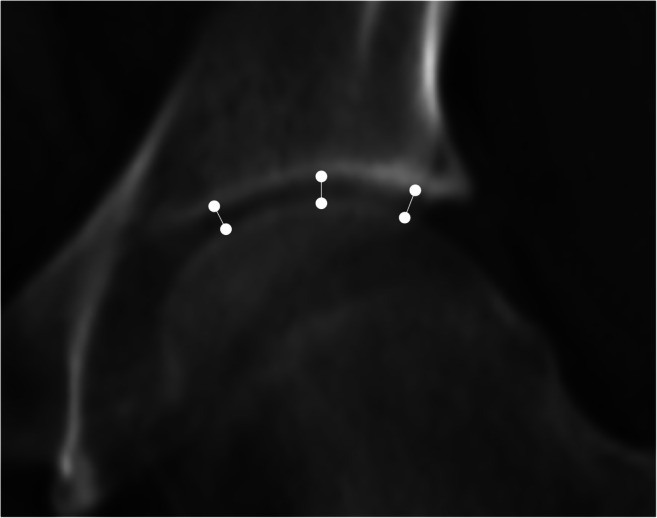
A 300-mm-thick coronal multiplanar reconstruction with an averaging of pixel intensities from a CT examination, as used to measure the joint space width. Location of the three sites of joint space width measurement is shown. From right to left: the superolateral, the apical, and the superomedial sites

Finally, we used the same radiographic projection to measure the JSW at three locations (apical, superomedial, and superolateral), located respectively at the zenith, the most medial and the most lateral aspects of the acetabular sourcil, respectively (Fig. 4).

### Statistical analyses

Right-left correlations were performed using the Spearman coefficient of correlation. Because of the strong correlation between the right and left hips for each patient, and in order to address the clustered nature of the data, the assessment of associations between the average size of ARO and other parameters was performed on 75 hips (one hip per patient randomly selected) [14]. The relationships between the dependent variable size of ARO and four categories of independent variables (patient-related parameters, hip JSW, and cam-type and pincer-type FAI morphology parameters) were successively tested using multiple regression.

The interobserver agreement was analyzed using intraclass correlation coefficients (ICC), with an absolute agreement model (systematic differences between readers considered relevant) for single measures (estimating the reliability of single ratings). The coefficients were interpreted as follows: ≤ 0 = poor, 0.01–0.20 = slight, 0.21–0.40 = fair, 0.41–0.60 = moderate, 0.61–0.80 = substantial, and ≥ 0.81 = almost perfect agreement. A *p* value of 0.05 was considered statistically significant for all analyses. Statistical tests were performed using R (R Core Team (2015)).

## Results

### Prevalence and topography of ARO

ARO was present in 96% (144 out of 150) (95% CI = [80.1; 100.0]) of the hips, with an average size of 1.78 mm (95% CI = [1.87; 3.08]). Table 1 reports the prevalence of labral ossification per age group. When considering the entire cohort, the number of hips with ARO was 33, 135, 136, and 127 for quadrants 1, 2, 3, and 4, respectively, while the average size was 0.21 mm, 1.31 mm, 3.30 mm, and 2.08 mm for quadrants 1, 2, 3, and 4, respectively.

**Table 1:** Prevalence of acetabular rim ossifications and average size of acetabular rim ossifications according to age

	Age range (years)				Total
	18-29	30-49	50-69	70-89	
Number of hips	38	40	52	20	150
Hips with acetabular rim ossifications^1^	34 (89.4%) [62.0; 100.0]	38 (95%) [67.2; 100.0]	52 (100%) [74.7; 100.0]	20 (100%) [61.1; 100.0]	144 (96%) [80.1; 100.0]
Average size of acetabular rim ossifications (mm)^2^	0.96 [0.71; 1.21]	1.46 [0.75; 1.28]	2.20 [1.43; 2.17]	2.48 [1.95; 2.47]	1.78 [1.87; 3.08]

^1^Data are raw numbers, followed by percentages in parentheses and 95% confidence intervals for percentages in brackets (%)

^2^Data are size followed by 95% confidence intervals for the size

### Right-left correlation of ARO

For all patients, there was a perfect correlation for the presence of ossifications between the right and left hips (three patients had no ossification on either side, and 72 had ossifications on both sides). The size of ARO was also statistically significantly correlated between the right and left hips (Spearman coefficient of correlation= 0.64 (95% CI = [0.46; 0.79]), *p* < 0.05)).

### Association between the size of ARO and patient-related parameters (age, gender, and BMI)

The size of ARO was not significantly associated with gender (*p* = 0.05) and BMI (*p* = 0.35) but was significantly associated with age (*p* < 0.0001).

### Association between the size of ARO and hip JSW

There was no significant association between the size of ARO and JSW measurements at either the apical, superomedial, or superolateral locations (all *p* ≥ 0.53).

### Association between the size of ARO and FAI morphology parameters

As reported in Table 2, there was no significant association between the size of ossifications and quantitative parameters (alpha angle and femoral head-neck offset) associated with cam-type morphology (all *p* ≥ 0.34).

**Table 2:** Association between the average size of ossifications and patient-related, and hip-related parameters

			Regression coefficient	*p* value
Patient-related parameters	BMI		0.02	0.35
	Age		0.04	< 0.001
	Gender		– 0.43	0.05
Hip-related parameters	Joint space width	Apical	0.17	0.53
		Superomedial	0.17	0.65
		Superolateral	0.06	0.82
	Cam morphology parameters	Alpha angle at 45°	0.00	0.81
		Offset	0.09	0.34
	Pincer morphology parameters	Acetabular version angle	– 0.01	0.83
		Lateral center-edge angle	0.02	0.56
		Acetabular index	0.03	0.53
		Crossover sign	0.26	0.41
		Posterior wall sign	– 0.25	0.47

There was no significant association between the size of ossifications and quantitative (lateral center-edge angle, acetabular index), or qualitative (crossover sign, posterior wall sign) parameters associated with pincer-type morphology (all *p* ≥ 0.41).

### Interobserver agreement

The interobserver agreement for the assessment of the average labral ossification size on 80 hips from 40 patients was substantial (ICC = 0.74 (95% CI = [0.69; 0.79])). The interobserver agreement for the measurement of JSW (240 measurements on 80 hips from 40 patients) was substantial (ICC = 0.71 (95% CI = [0.64; 0.77])). The interobserver agreement for the assessment of parameters associated with FAI morphology on 80 hips from 40 patients was substantial to almost perfect (ICC ranging from 0.76 (95% CI = [0.65; 0.84]) to 0.96 (95% CI = [0.94; 0.97]), except for the alpha angle for which it was fair (ICC = 0.56 (95% CI = [0.38; 0.69])).

## Discussion

In this study, our main findings were that (1) the prevalence of ARO is high in asymptomatic hips with no imaging sign of osteoarthritis, (2) there is a right-left side association both for the presence of ARO and their size, (3) age is the only patient-related parameter that is associated with ARO, and (4) there is no association of ARO with hip-related morphological parameters, including quantitative and qualitative parameters descriptive of FAI morphology.

There is only limited literature on the prevalence of ARO in normal hips [5, 7]. To the best of our knowledge, this is the first study on the prevalence of ARO in asymptomatic hips with no signs of osteoarthritis at CT. We have shown that 96% (95% CI = [80.1; 100.0]) of these hips had ARO. ARO were present at all ages, and their prevalence increased with age. In a previous cadaveric study using macroscopic evaluation and fine detail radiographs of 365 acetabula, Byers et al found that ossification at the attachment site of the labrum around the acetabular rim may affect subjects at an early age, increased with age, and affected around 50% of people over the age of 50 [2]. The higher prevalence of ARO in our population of asymptomatic hips is likely related to the different methods used to detect them. While macroscopic assessment might have missed some smaller or deeply located ossifications, select fine detail radiographs are intrinsically limited for a thorough assessment of the acetabular rim. As previously suggested, among clinically available modalities, CT is likely the method of choice for a thorough assessment of ossifications of the acetabular rim, both in terms of resolution and contrast [7].

The origin of ARO is unclear. Several of our findings suggest that ARO represent an entity distinct from osteophytes. First, ARO were highly prevalent, and occasionally with a fairly large size, in a cohort of asymptomatic hips from which all joints with other signs of osteoarthritis—including any osteophytes at other locations in the joint, subchondral bone changes, and joint space narrowing—had been excluded. Second, the fact that younger asymptomatic adults are frequently affected (up to 90% between the ages of 18 and 29, and over 95% afterward) suggests that the process of ossification is unrelated to the development of osteoarthritis. These findings are in line with previous studies. In their cadaveric study, Byers et al found ARO in young subjects, even before 19 years of age [2]. Corten et al showed in a series of 20 patients who had undergone hip surgery that the presence of ARO was not associated with increased joint degeneration, concluding that this process of ossification is distinct from osteophyte formation [1]. Byrd et al, in an arthroscopic series of 56 hips with and 56 hips without labral ossifications, found that none of the patients with labral ossifications had acetabular osteophytes or significant cartilage damage, further supporting the hypothesis that ARO correspond to ossifications of the acetabular labrum and exist distinctly from acetabular osteophytes [7].

In practice, in the absence of other signs of hip osteoarthritis, the interpretation of ARO and the differentiation between labral ossifications and osteophytes is challenging. According to some authors, a diagnostic clue characteristic of labral ossifications, which is not present in osteophytes, is the presence of a notch at the interface between the acetabulum and the ossified labrum, likely corresponding to the chondrolabral recess (Fig. 3d) [4]. However, in our cohort, this sign was rarely present. In practice, because of the high prevalence of ARO in asymptomatic non-osteoarthritic hips, the sole presence of an ARO should not be interpreted as an osteophyte and osteoarthritis should only be considered if other clinical or imaging signs of the disease are present. In particular, the presence at imaging of osteophytes at other locations (specifically marginal osteophytes of the femoral head, and of the fovea capitis) is a useful diagnostic clue, along with other cardinal signs of osteoarthritis.

Interestingly we have shown no association between the size of ARO and any of the patient-related parameters except for age, which was in line with the findings by Byers et al as discussed above [2]. Furthermore, there was no association between the size of ARO and any of the parameters describing hip morphology, either hip JSW, or quantitative and qualitative parameters associated with FAI morphology. Previous studies have suggested that ARO might be the cause or the consequence of FAI [1, 7]. By studying 148 hips treated for FAI, and showing a higher prevalence of ARO in hips with coxa profunda vs. hips without coxa profunda (29% vs. 8% respectively), Corten et al postulated that the ossification originating at the subperiosteal region of the outer acetabular rim was a consequence of pre-existing pincer-type FAI [1]. However, that study suffered some limitations. First, ARO were assessed using radiographs and MRI, and considered present when visible on both modalities, which is likely to have led to an underestimation of the prevalence of these ossifications compared to an assessment using CT. Second, and more importantly, the study was limited to symptomatic hips and it is not clear whether asymptomatic hips would show a lower prevalence of ARO. Our results confirm that ARO commonly exist in the absence of FAI morphology. Byrd et al have compared two groups of patients who had undergone arthroscopy for pincer-type FAI, one with and one without labral ossifications [7]. They have concluded that patients with labral ossifications represent a unique subset of pincer-type FAI, more likely to be older, female, and with more severe symptoms. While it is tempting in view of these previous reports to consider the presence of ARO as an imaging sign of pincer-type impingement, as has been also suggested for isolated mineralization of the acetabular labrum, our findings show that the presence of labral ossification should not be interpreted as a sign of pathology, due to their almost ubiquitous presence in asymptomatic hips [15]. Finally, it should be reminded and emphasized that the diagnosis of FAI requires both the presence of clinical signs and FAI morphology at imaging. As reported previously, the diagnosis of FAI should not be made on the sole presence of FAI morphology [13, 16].

The distribution of ARO around the acetabular rim might give insight into their pathophysiology. Ossifications were more frequently present and larger in the posterior quadrants compared to the anterior quadrants. Biomechanical studies have shown that contact forces in the hip joint during routine activities such as walking at different speeds or going up and down the stairs predominate anteromedially [17]. Therefore, it is unlikely that ARO develop as a result of mechanical loading. The absence of any association between ARO and the parameters related to the morphology of the hip, which may influence mechanical constraints, also suggests that the latter have little role in this ossification process. Further studies are needed in order to understand the pathophysiology of ARO.

The findings of this study must be considered in the context of its limitations. First, the selection of patients was done through a questionnaire, and clinical examination of the hip was not available. Second, we had no histological correlation, but this was beyond the scope of our study. As discussed above, we rather aimed at a thorough examination of the acetabular rim for the presence and size of ossifications, and CT is the modality of choice to do this in a clinical setting [7]. Third, the imaging was acquired on three different scanners, which could have influenced image interpretation. However, this potential bias was limited by the fact that all three devices were from the same manufacturer, and image quality was optimized by the same team of radiologists. Fourth, the population was limited in size and was derived from a single institution. Selection bias may be present in terms of patient ethnicity and lifestyle/level of activity, despite the fact that this was a large university hospital covering a diverse population pool. Finally, we did not assess the intraobserver agreement for the assessment of the ossification or measurements. But this is not required when interobserver agreement is high, as was the case in our study [18].

In conclusion, we have shown that ARO are highly prevalent in asymptomatic, non-osteoarthritic hips and that they are not correlated with any patient- or hip-related parameters except for age. In particular, there is no association between ARO and any of the quantitative or qualitative parameters described for FAI morphology. Taken together, our findings suggest that ARO exist independently from osteophytes in adult hips at all ages and should not be interpreted as a pathological finding. Care should be taken to avoid overdiagnosis of osteoarthritis or FAI morphology in the sole presence of ARO.

## Abbreviations

ARO: Acetabular rim ossifications
BMI: Body mass index
CT: Computed tomography
FAI: Femoroacetabular impingement
ICC: Intraclass correlation coefficients
JSW: Joint space width
PACS: Picture archiving and communication system

## Declarations

### Guarantor

The scientific guarantor of this publication is Dr. Patrick Omoumi, Lausanne University Hospital.

### Statistics and biometry

Hugo Babel kindly provided statistical advice for this manuscript.

### Study subjects or cohorts overlap

Our sample population is taken from the same cohort of patients as two previously published studies, and partly (about 80%) overlaps with the sample populations of these studies (M. Saucier Lepage et al Femoroacetabular impingement: normal values of the quantitative morphometric parameters in asymptomatic hips. *European Radiology* 2014 24:1707–1714 and P. Omoumi et al Anatomic Features Associated with femoroacetabular impingement are equally common in hips of old and young asymptomatic individuals without CT signs of osteoarthritis. *AJR* y 2014; 202:1078-1086). However, the scope of this study is entirely unrelated to these previous studies, and our results have never been published or presented so far.

### Methodology

• prospective

• observational

• performed at one institution
